# Identifying high-risk patients having ERCP as a day surgery with an online prediction platform: Multicohort validation of a machine learning model

**DOI:** 10.1055/a-2733-1387

**Published:** 2025-12-16

**Authors:** Boru Jin, Yi Wang, Xu Zhang, Jinyu Zhao, Wangping He, Kecheng Jin, Zhen Liu, Ruyang Zhong, Yuhu Ma, Chunlu Dong, Yanyan Lin, Xiaoliang Zhu, Kexiang Zhu, Lei Zhang, Ping Yue, Shuyan Li, Jinqiu Yuan, Xun Li, Wenbo Meng

**Affiliations:** 1 12426 Lanzhou University, Lanzhou, China; 2 91589 Endoscopic diagnosis and treatment center, Gansu Provincial Hospital, Lanzhou, China; 3 12426 Anesthesiology, Lanzhou University, Lanzhou, China; 4 117741 Lanzhou University First Clinical Medical College, Lanzhou, China; 5 117741 The Fourth Department of General Surgery, Lanzhou University First Hospital, Lanzhou, China; 6 117741 The Third Department of General Surgery, Lanzhou University First Hospital, Lanzhou, China; 7 117741 The Second Department of General Surgery, Lanzhou University First Hospital, Lanzhou, China; 8 38044 School of Medical Information and Engineering, Xuzhou Medical University, Xuzhou, China; 9 543160 Clinical Research Center, Big Data Center, The Seventh Affiliated Hospital Sun Yat-sen University, Shenzhen, China; 10 117741 Lanzhou University First Hospital, Lanzhou, China

**Keywords:** Pancreatobiliary (ERCP/PTCD), Quality and logistical aspects, Performance and complications, GI surgery

## Abstract

**Background and study aims:**

This study aimed to develop a clinical prediction model to assess the 24-hour post-ERCP complication risk in patients with common bile duct stones (CBDs), guiding clinical decision-making for ERCP as a day surgery.

**Patients and methods:**

Retrospective data from The First Hospital of Lanzhou University (2010–2019) and prospective multicenter data on post-ERCP complications (2020–2023) were collected and registered on ClinicalTrials.gov (NCT04234126, NCT04242394). The ADASYN method was used for dataset balancing. Machine learning algorithms, including KNN, XGBoost, RF, SVM, and NB, were compared with traditional models. External validation was performed with retrospective data from other ERCP centers (2015–2017) and The First Hospital of Lanzhou University (2019–2020), with registration under NCT02510495. The optimal model was selected based on the ROC curve (AUC), and an online prediction tool was developed.

**Results:**

A logistic regression (LR) model incorporating seven feature variables—mechanical lithotripsy, pancreatic duct cannulation, bile duct dilation, residual stones, white blood cell count, alanine aminotransferase (ALT) level, and pancreatic duct stent placement—was identified as the optimal model, The model yielded specificity, sensitivity, accuracy, and AUC values of 0.835, 0.655, 0.807, and 0.819 in the external validation set, with a second external validation set providing additional results of 0.799, 0.714, 0.784, and 0.805. Patients were stratified into high- and low-risk groups. An online calculator was developed ( https://borujin.shinyapps.io/dynnomapp/ ).

**Conclusions:**

The results indicate that the proposed LR model, utilizing the top seven risk factors, could serve as an effective tool for predicting occurrence of complications in day surgery.

## Introduction

Bile duct stones are a common gastrointestinal disease; in China, incidence of this condition is 9.5% to 21.3%. Endoscopic retrograde cholangiopancreatography (ERCP) is an important technique for treating common bile duct (CBD) stones 1 . Due to its minimal invasiveness, ERCP allows patients without complications to resume eating and be discharged quickly. However, patients who do develop complications naturally require longer hospitalization. Therefore, identifying and predicting complications of ERCP and implementing stratified management strategies are highly important.

Day surgery management is defined as a hospitalization model in which admission, diagnosis, surgery, and discharge are accomplished within 24 hours 2 . Complications that could develop following ERCP have jointly become the primary obstacle to development of its corresponding day surgery model. These complications mainly include post-ERCP pancreatitis (PEP), cholangitis, cholecystitis, bleeding, and perforation 3 4 .

This study aimed to develop a predictive model for complications following day-surgery ERCP by employing multicenter clinical data to establish and validate a 24-hour postoperative risk assessment tool 1 .

## Patients and methods

### Study design

**Fig. 1:**
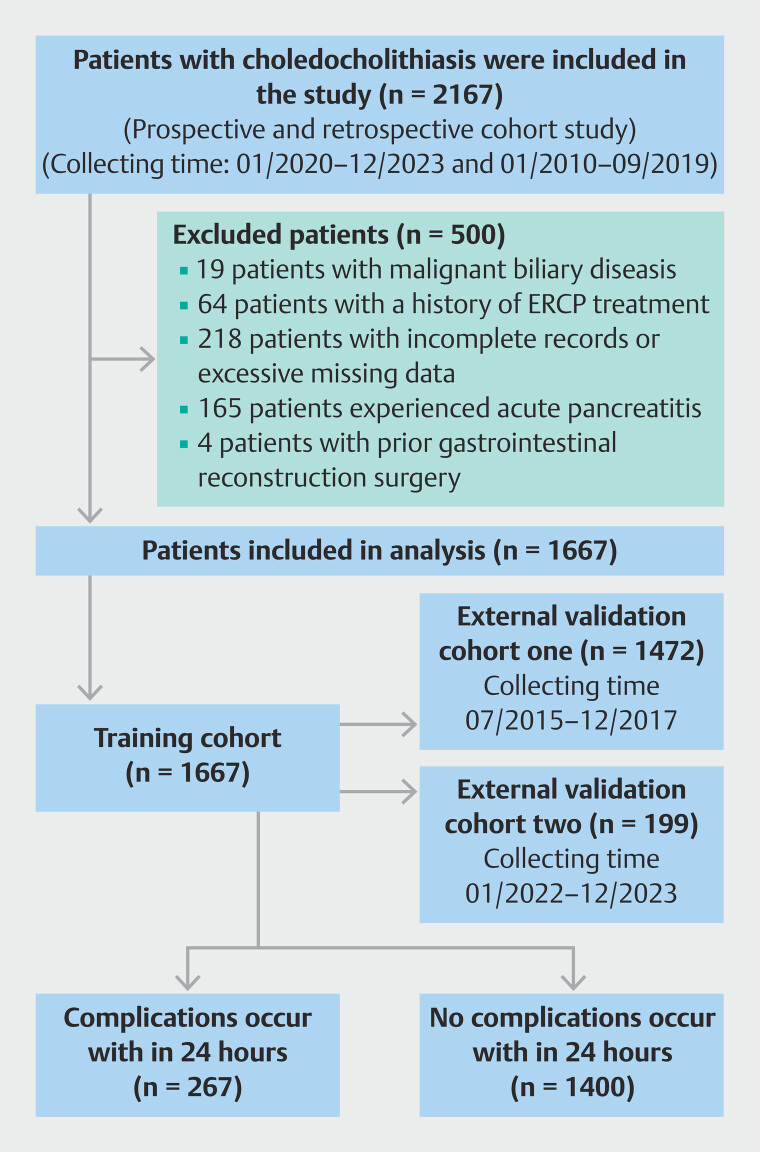
Flowchart of model patient enrollment.

Inclusion criteria were age ≥ 18 years and definitive diagnosis of CBD stones. Exclusion criteria were: 1) presence of malignant biliary diseases; (2) history of ERCP treatment; 3) incomplete or substantially missing data; 4) preoperative acute pancreatitis; 5) history of gastrointestinal reconstruction surgery; and 6) history of cholecystectomy. A flowchart was designed ( Fig. 1 ). Patients with prior ERCP were excluded to ensure a naïve papilla for the current procedure, although not excluding those with other prior papillary interventions. We excluded patients with prior cholecystectomy mainly to prevent confusion between post-cholecystectomy symptoms and genuine ERCP-related complications. Furthermore, this exclusion maintains cohort homogeneity, ensuring that the prediction model remains specifically applicable to choledocholithiasis patients with intact gallbladders. The overall mind map can be found in the supplementary materials ( **Supplementary Fig. 1** ). [Heading 2]Diagnostic criteria for post-ERCP complications

Diagnostic criteria and methodology for grading severity of ERCP-related postoperative complications in this study were obtained from the European Society of Gastrointestinal Endoscopy (ESGE) guidelines 5 . PEP was defined based on Cotton's mild grading criteria 6 : new or worsening abdominal pain combined with an increase in amylase or lipase levels to more than three times the standard value lasting for over 24 hours and requiring hospitalization or an extended hospital stay of 2 to 3 days. Post-ERCP cholecystitis(PEC) was defined based on the Tokyo Guidelines 7 : A. Local signs of inflammation: 1) Murphy's sign; 2) right upper quadrant mass/pain/tenderness; B. Systemic signs of inflammation: 1) fever; 2) elevated C-reactive protein (CRP) levels; and 3) leukocytosis; C. Imaging findings: Characteristic imaging findings of acute cholecystitis identified on abdominal ultrasound, computed tomography (CT), or magnetic resonance imaging (MRI) of the biliary tract. Suspected diagnosis: One item from A + one item from B. Confirmed diagnosis One item from A + one item from B + item C. Post-ERCP cholangitis was defined as newly elevated body temperature and gamma-glutamyl transferase (γ-GT) levels; specifically, a body temperature of ≥ 38°C persisting for more than 24 hours, accompanied by biliary obstruction, defined as γ-GT levels exceeding the normal range 8 . Post-ERCP bleeding was defined as hematemesis, melena, or a decrease in hemoglobin of at least 2 g/dL 8 . Post-ERCP perforation was defined as presence of extraluminal gas or luminal contents confirmed by imaging 8 . The occurrence of any of the above complications within 24 hours post-ERCP was considered a positive outcome, and each patient could have multiple positive outcomes if they presented with different complications.

### Procedures

A total of 42 variables were included in the feature selection process on the basis of recent, relevant literature on ERCP-related complications and clinical experience in treatment of CBD stones with ERCP. The following data were collected: 1) general information, such as demographic characteristics, including sex, age, and body mass index (BMI), as well as comorbidities; 2) laboratory indicators, including white blood cell (WBC) count, hemoglobin, platelet count, serum alkaline phosphatase (ALP), alanine aminotransferase (ALT), aspartate aminotransferase (AST), measured both preoperatively and at 12, 24, and 48 hours postoperatively; 3) vital signs, including body temperature and heart rate, and postoperative clinical manifestations, such as abdominal distension, vomiting, hematemesis, or melena; 4) preoperative and postoperative imaging examination data, including those from abdominal ultrasound, magnetic resonance cholangiopancreatography (MRCP), and abdominal CT; and 5) intraoperative factors, such as operative time, guidewire entry into the pancreatic duct, difficult cannulation, intraoperative bleeding, and residual stones (defined as persistent filling defects observed on completion cholangiography after standard stone extraction procedures).

### Statistical analysis

Statistical analyses were performed with SPSS v.19.0 and RStudio (version 3.4.2). Variables with more than 10% missing data were excluded and multiple linear imputation was applied to handle any missing values for the remaining eligible data. Continuous variables with a normal distribution are expressed as means ± standard deviations (SDs), and intergroup differences were analyzed with the *t* test. Variables that did not follow a normal distribution are described using medians and interquartile ranges (IQRs), and between-group comparisons were conducted with the Mann-Whitney U test. Categorical variables are expressed as frequencies (percentages) and between-group differences were assessed with the Pearson chi-square test. Variables with an absolute Spearman correlation coefficient > 0.8 and a variance inflation factor (VIF) > 10 were defined as having strong multicollinearity. On the basis of the correlation coefficient distribution plot ( **Supplementary Fig. 2** ), four highly correlated variables were removed, leaving 38 variables subject to subsequent regression analysis.

The Adaptive Synthetic Sampling (ADASYN) algorithm was used to oversample the minority class. ADASYN generates synthetic samples on the basis of characteristics of the local distribution of nearest neighbors 9 10 11 . It is particularly effective for handling minority class samples for which differentiation can be difficult. ADASYN offers a balance of high-quality sample generation and noise control. After the ADASYN algorithm was applied, frequency distributions for continuous and categorical variables and scatterplots of the overall data distribution were visualized ( **Supplementary Fig. 3** ).

### Variable selection and modelling

We conducted a comprehensive comparison of multiple machine learning algorithms and traditional statistical models to ensure methodological rigor. This evaluation served two key purposes: to assess the capacity of complex models to capture nonlinear relationships and to verify the robustness of logistic regression with limited datasets. Through this thorough assessment, we established the optimal modelling approach.

On the basis of all the clinical features, five machine learning models were used for the initial predictions: K-nearest neighbors (KNN), support vector machine (SVM), naïve Bayes (NB), extreme gradient boosting (XGBoost), and random forest (RF).The entire dataset was followed by 10-fold cross-validation to determine average predictive performance of the models. Model evaluation metrics included area under the receiver operating characteristic (ROC) curve (AUC), accuracy (ACC), sensitivity (SEN), specificity (SPE), precision (PRE), and Brier score and were used to identify the initial optimal model. To further refine the model, the five machine learning models were subjected to five different variable importance ranking methods: the Boruta algorithm, permutation importance, the conditional probability method, SHapley Additive exPlanations (SHAP) visualization analysis, and Gini importance. For each model ranking method, variables were incorporated step-by-step, and optimal hyperparameters were tuned through 10-fold cross-validation.

The average AUC from the 10-fold cross-validation sets was calculated and the number of variables corresponding with the maximum AUC for each of the five models was recorded. These optimal sets of variables were subsequently applied to the external test set to identify the best-performing machine learning model. To comprehensively identify the best-performing model, we also developed various logistic regression and LASSO regression models. Logistic regression analysis was performed on all feature variables via univariable preselection of features followed by multivariable modelling to identify independent risk factors. In addition, forward and backward stepwise regression methods were employed and compared with the univariable followed by multivariable approach LASSO regression was applied to determine the optimal feature variables for modelling. The model with the best performance metrics was selected. Utility and clinical value of the model were subsequently assessed. When analyzing continuous variables, we employed restricted cubic splines (RCS) to identify potential nonlinear relationships and modeled variables based on knot distribution. According to the Youden index, the optimal cut-off value was identified and risk stratification was performed. A dynamic nomogram was constructed and a corresponding web-based real-time prediction tool was developed to facilitate practical application of the model.

## Results

### Baseline patient characteristics

This study included 1,667 patients diagnosed with CBD stones who underwent ERCP for stone extraction between 2010 and 2019 and 2020 and 2023. The cohort comprised 1,400 patients (84.0%) without postoperative complications within 24 hours and 267 patients (16.0%) with complications, demonstrating significant class imbalance ( Table 1 ).

**Table:** **Table 1** Original baseline characteristics table.

**Variables**	**Overall**	**No complications within 24 hours**	**Complications within 24 hours**	***P* value **
	**N = 1667**	**N = 1400**	**N = 267**	
Gender (%)				
Female	748 (44.9)	629 (44.9)	119 (44.6)	0.914
male	919 (55.1)	771 (55.1)	148 (55.4)	
Common bile duct dilatation (%)				
No	519 (31.1)	457 (32.6)	62 (23.2)	0.002
Yes	1148 (68.9)	943 (67.4)	205 (76.8)	
Common bile duct obstruction (%)				
No	1110 (66.6)	952 (68.0)	158 (59.2)	0.005
Yes	557 (33.4)	448 (32.0)	109 (40.8)	
Multiple stones (%)				
No	667 (40.0)	568 (40.6)	99 (37.1)	0.286
Yes	1000 (60.0)	832 (59.4)	168 (62.9)	
Bile duct stent (%)				
No	1018 (61.1)	861 (61.5)	157 (58.8)	0.407
Yes	649 (38.9)	539 (38.5)	110 (41.2)	
Coronary heart disease (%)				
No	1600 (96.0)	1346 (96.1)	254 (95.1)	0.44
Yes	67 (4.0)	54 (3.9)	13 (4.9)	
Chronic lung disease (%)				
No	1631 (97.8)	1375 (98.2)	256 (95.9)	0.016
Yes	36 (2.2)	25 (1.8)	11 (4.1)	
Intraoperative bleeding (%)				
No	1522 (91.3)	1283 (91.6)	239 (89.5)	0.258
Yes	145 (8.7)	117 (8.4)	28 (10.5)	
Pancreatic duct stent (%)				
No	1581 (94.8)	1334 (95.3)	247 (92.5)	0.06
Yes	86 (5.2)	66 (4.7)	20 (7.5)	
Difficult cannulation (%)				
No	1520 (91.2)	1285 (91.8)	235 (88.0)	0.046
Yes	147 (8.8)	115 (8.2)	32 (12.0)	
Pancreatic duct cannulation (%)				
No	1472 (88.3)	1262 (90.1)	210 (78.7)	< 0.001
Yes	195 (11.7)	138 (9.9)	57 (21.3)	
Epinephrine spray(%)				
No	1507 (90.4)	1274 (91.0)	233 (87.3)	0.058
Yes	160 (9.6)	126 (9.0)	34 (12.7)	
Electrocautery hemostasis (%)				
No	1662 (99.7)	1399 (99.9)	263 (98.5)	< 0.001
Yes	5 (0.3)	1 (0.1)	4 (1.5)	
Young female (%)				
No	1483 (89.0)	1244 (88.9)	239 (89.5)	0.754
Yes	184 (11.0)	156 (11.1)	28 (10.5)	
Endoscopic sphincterotomy (%)				
No	195 (11.7)	166 (11.9)	29 (10.9)	0.643
Yes	1472 (88.3)	1234 (88.1)	238 (89.1)	
Endoscopic papillary balloon dilation (%)				
No	1204 (72.2)	1026 (73.3)	178 (66.7)	0.027
Yes	463 (27.8)	374 (26.7)	89 (33.3)	
Periampullary duodenal diverticulum (%)				
No	1306 (78.3)	1101 (78.6)	205 (76.8)	0.498
Yes	361 (21.7)	299 (21.4)	62 (23.2)	
Mechanical lithotripsy (%)				
No	1416 (84.9)	1211 (86.5)	205 (76.8)	< 0.001
Yes	251 (15.1)	189 (13.5)	62 (23.2)	
Nasobiliary drainage (%)				
No	598 (35.9)	499 (35.6)	99 (37.1)	0.654
Yes	1069 (64.1)	901 (64.4)	168 (62.9)	
Intraoperative residual stones (%)				
No	1352 (81.1)	1155 (82.5)	197 (73.8)	0.001
Yes	315 (18.9)	245 (17.5)	70 (26.2)	
Hypertension (%)				
No	1314 (78.8)	1103 (78.8)	211 (79.0)	0.93
Yes	353 (21.2)	297 (21.2)	56 (21.0)	
Diabetes mellitus (%)				
No	1535 (92.1)	1295 (92.5)	240 (89.9)	0.147
Yes	132 (7.9)	105 (7.5)	27 (10.1)	
Age (median [IQR])	62.00 (49.00–73.00)	62.00 (49.00–73.00)	63.00 (49.00–73.00)	0.948
MRCPDI (median [IQR])	10.00 (9.00–13.00)	10.00 (8.28–13.00)	11.00 (9.00–13.00)	0.449
DBIL (median [IQR])	16.00 (5.75–55.25)	15.35 (5.70–53.15)	22.60 (5.90–62.80)	0.128
Height (median [IQR])	1.65 (1.60–1.72)	1.65 (1.60–1.72)	1.65 (1.60–1.72)	0.968
TG (median [IQR])	1.34 (0.94–2.05)	1.35 (0.95–2.04)	1.29 (0.86–2.12)	0.226
Operative time (median [IQR])	40.00 (28.00–50.00)	40.00 (28.00–50.00)	40.00 (30.00–50.00)	0.688
BILMAX (median [IQR])	0.80 (0.50–1.00)	0.80 (0.50–1.00)	0.80 (0.50–1.00)	0.319
HGB (median [IQR])	139.00 (125.00–153.00)	140.00 (125.00–153.00)	139.00 (126.00–151.00)	0.949
PLT (median [IQR])	180.00 (128.00–234.00)	184.00 (131.75–235.00)	165.00 (116.50–233.00)	0.017
ALB (median [IQR])	40.20 (36.00–44.10)	40.20 (36.00–44.20)	40.30 (36.15–44.00)	0.916
BMI (median [IQR])	22.86 (20.70–25.25)	22.77 (20.70–25.26)	23.01 (20.66–25.01)	0.796
WBC (median [IQR])	6.05 (4.74–8.66)	5.94 (4.67–8.20)	7.41 (5.06–12.12)	< 0.001
Blood amylase (median [IQR])	58.00 (43.00–86.00)	58.70 (44.00–86.00)	57.00 (41.65–84.25)	0.916
ALP (median [IQR])	196.30 (123.00–350.00)	196.00 (123.00–347.00)	202.00 (119.90–377.30)	0.742
YGT (median [IQR])	293.50 (113.25–574.95)	294.35 (110.22–556.22)	289.20 (126.60–648.45)	0.114
ALT (median [IQR])	99.00 (39.00–223.00)	96.70 (38.00–218.25)	104.00 (43.50–257.00)	0.162
ALB, albumin; ALP, alkaline phosphatase; ALT, alanine aminotransferase; AST, aspartate aminotransferase; BILMAX, biliary stone maximum diameter; BMI, body mass index; DBIL, direct serum bilirubin; MRCPDI, inner duct diameter on magnetic resonance cholangiopancreatography; PLT, platelet; TG, triglycerides; WBC, white blood cell; YGT, gamma-glutamyl transferase.				

Following ADASYN implementation, the balanced dataset contained 2,201 patients while preserving the original data characteristics and distributions ( **Supplementary Fig. 3** ). The complication group proportion increased to 36.4% (801/2,201), significantly enhancing the model's ability to identify minority class cases ( **Supplementary Table 1** ).

### Primary Outcomes

The original data analyses revealed several significant findings. CBD dilation was more frequent in the complication group than in the non-complication group (76.8% vs. 67.4%, *P* = 0.002), as was CBD obstruction (40.8% vs. 32.0%, *P* = 0.005). Pancreatic duct cannulation (21.3% vs. 9.9%, *P* < 0.001) and difficult biliary cannulation (12.0% vs. 8.2%, *P* = 0.046) were both significantly associated with postoperative complications. Patients with chronic lung disease had higher complication rates (4.1% vs. 1.8%, *P* = 0.016), as did those requiring electrocautery hemostasis (1.5% vs. 0.1%, *P* < 0.001). Endoscopic papillary balloon dilation (33.3% vs. 26.7%, *P* = 0.027) and mechanical lithotripsy (23.2% vs. 13.5%, *P* < 0.001) were performed more frequently in the complication group. WBC count was significantly elevated in the complication group (median 9.47 vs 5.94, *P* < 0.001). The proportion of intraoperative residual stones was significantly greater in the complication group (26.2% vs. 17.5%, *P* = 0.001) ( Table 1 ).

### Secondary outcomes

Ten core predictors maintained significance ( *P* < 0.05) across both datasets, demonstrating consistent associations with complications. ADASYN identified new predictors: pancreatic duct stent ( *P* = 0.033), epinephrine spray ( *P* = 0.049), inner duct diameter on magnetic resonance cholangiopancreatography ( *P* = 0.008), gamma-glutamyl transferase ( *P* = 0.002), direct serum bilirubin ( *P* = 0.002), platelet count ( *P* = 0.005), and ALT ( *P* < 0.001), all of which were clinically plausible for biliary procedures.

### Full-feature machine learning model

The dataset and all feature variables were used in the construction of the KNN, XGBoost, RF, SVM, and NB models for initial comparison via 10-fold cross-validation. The models were evaluated with metrics including AUC, ACC, SEN, SPE, PRE, and Brier score. AUC values for the five models were as follows: KNN 0.847, XGBoost 0.879, RF 0.902, NB 0.765, and SVM 0.837. Among these models, the RF model demonstrated the best overall performance ( Table 2 ).

**Table:** **Table 2** Details of models constructed with the training set via 10-fold cross-validation.

**Factors**	**AUC**	**Accuracy**	**Sensitivity**	**Specificity**	**Brier Score**	**Precision**
XGB mean	0.879	0.812	0.677	0.889	0.131	0.828
XGB SD	0.003	0.004	0.009	0.008	0.001	0.003
RF mean	0.902	0.840	0.682	0.930	0.130	0.837
RF SD	0.003	0.005	0.011	0.006	0.001	0.004
NB mean	0.765	0.652	0.071	0.985	0.324	0.650
NB SD	0.010	0.006	0.024	0.005	0.010	0.005
KNN mean	0.847	0.793	0.802	0.787	0.144	0.874
KNN SD	0.009	0.011	0.016	0.011	0.007	0.010
SVM mean	0.837	0.793	0.597	0.905	0.144	0.797
SVM SD	0.006	0.009	0.012	0.010	0.004	0.005
XGB, extreme gradient boosting; RF, random forest; NB, naive Bayes; KNN, k-nearest neighbors; SVM, support vector machine; SD, standard deviation; BS, Brier score = Σ(Predicted Probability – Actual Outcome)²/n. BS ranges from 0 to 1 and is used to evaluate performance of probabilistic models.						

### Machine learning-based refined variable selection and hyperparameter optimization

Variable importance rankings for the RF, KNN, SVM, XGBoost, and NB models were assessed via five methods ( **Supplementary Fig. 4a, Supplementary Fig. 4b, Supplementary Fig. 4c, Supplementary Fig. 4d, Supplementary Fig. 4e** , respectively). Among the five ranking methods, WBC, pancreatic duct cannulation, and ALT consistently demonstrated stable and significant contributions to the predictive performance across all models. The optimal number of features for model construction was as follows: RF with 12 features, XGBoost with 13 features, KNN with 19 features, SVM with 12 features, and NB with 14 features. The XGBoost (AUC = 0.927) and RF (AUC = 0.916) models achieved the highest AUCs. In contrast, KNN, SVM, and NB performed less effectively, with AUCs of 0.826, 0.840, and 0.765, respectively ( **Supplementary Fig. 4f** ). At each step, 10-fold cross-validation and hyperparameter tuning were performed on the overall dataset ( **Supplementary Fig. 5** , **Supplementary Table 2** ).

### Logistic regression and LASSO regression modelling

**Fig. 2:**
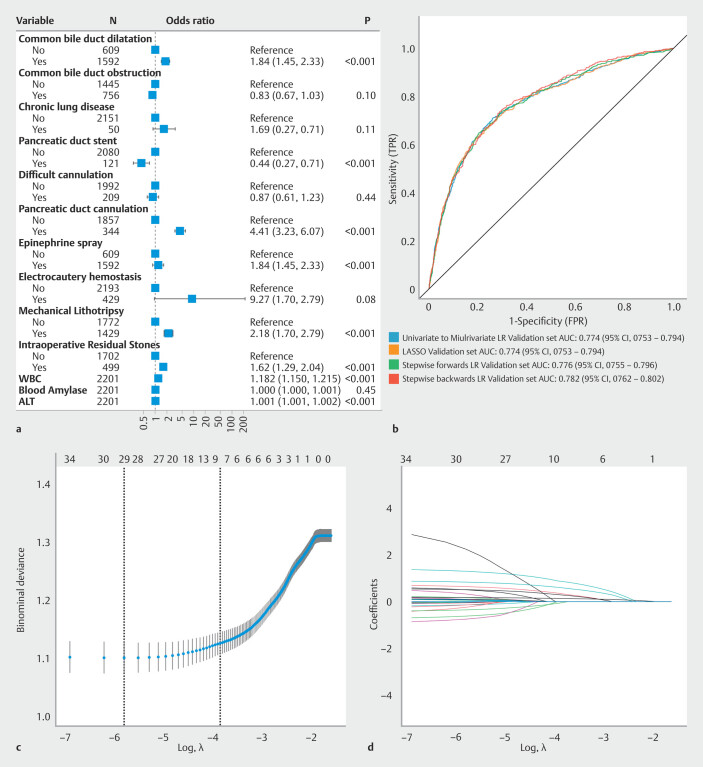
Variable selection and modelling flowchart for various logistic regression and LASSO regression methods. a Forest plot for univariate to multivariate logistic regression. b Mean AUC values of various logistic regression and LASSO regression models following 10-fold cross-validation. c Cross-validation plot for LASSO regression; Final variable selection; d Graph of coefficient paths for LASSO regression.

Univariate to multivariate logistic regression analysis revealed seven significant features, including seven independent risk factors ( Fig. 2 **a** ) – bile duct dilation ([OR] 1.84; 95% CI 1.45–2.33; *P* < 0.001), pancreatic duct cannulation (OR 4.41; 95% CI 3.23–6.07; *P* < 0.001), mechanical lithotripsy (OR 2.18; 95% CI 1.70–2.79; *P* < 0.001), intraoperative stone residue (OR 1.62; 95% CI 1.29–2.04; *P* < 0.001), WBC count (OR 1.182; 95% CI 1.150–1.215; *P* < 0.001), and ALT (OR 1.001; 95% CI 1.001–1.002; *P* < 0.001)–and one independent protective factor: pancreatic duct stenting (OR 0.44; 95% CI 0.27–0.71; *P* < 0.001). These models built with these selected features achieved average AUCs of 0.774 in the corresponding validation sets. The AUC of the forward method and the backward method were 0.776 and 0.782.( Fig. 2 **b** ). Use of LASSO regression with lambda.1se regularization parameter yielded seven key variables ( Fig. 2 **c,d** ).

### External test set and final model

**Fig. 3:**
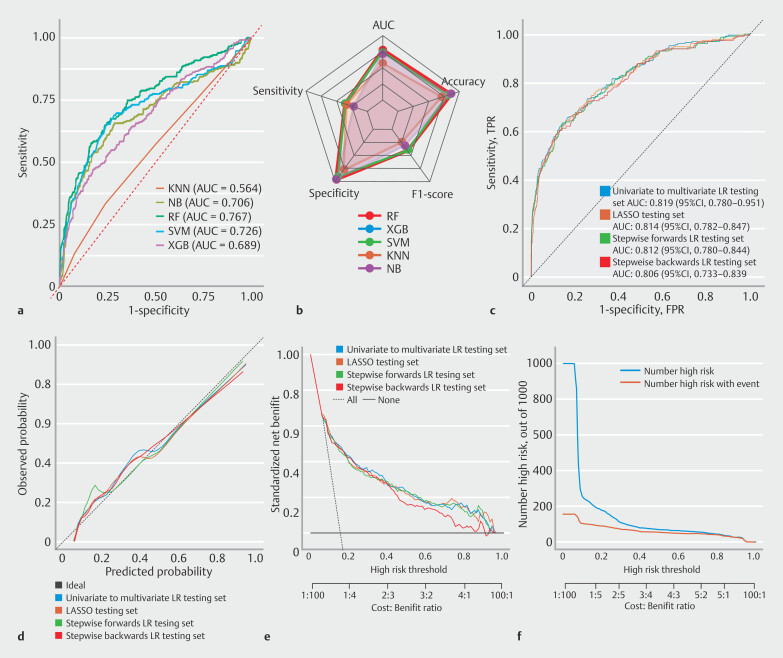
ROC curve and clinical evaluation metrics for external validation set. **a** ROC curves of various machine learning models in the test set. **b** Performance evaluation metrics of multiple models. **c** ROC curves of the various logistic regression and LASSO models in the test set. **d** Calibration curves of the various logistic regression and LASSO models in the test set. Clinical calibration curves revealed that models had good calibration performance. However, the univariate to multivariate model aligned more closely with the ideal line. **e** Decision curves of the various logistic regression and LASSO models in the test set. Clinical decision curve analysis was used to evaluate the clinical net benefit of the models at different risk thresholds. The Univariate to Multivariate logistic regression model consistently achieved higher net benefits across multiple risk thresholds (0.1–0.95). **f** Clinical impact curve of the univariate to multivariate (best model) logistic regression model. The clinical impact curve for the univariate to multivariate logistic regression model revealed that at low-risk thresholds (0–0.1), the model identified a greater number of high-risk individuals, albeit with a higher false-positive rate. As the threshold increased to medium levels (0.1–0.8), accuracy of the model predictions improved, and at high thresholds (0.8–1), prediction precision was maximized.

**Fig. 4:**
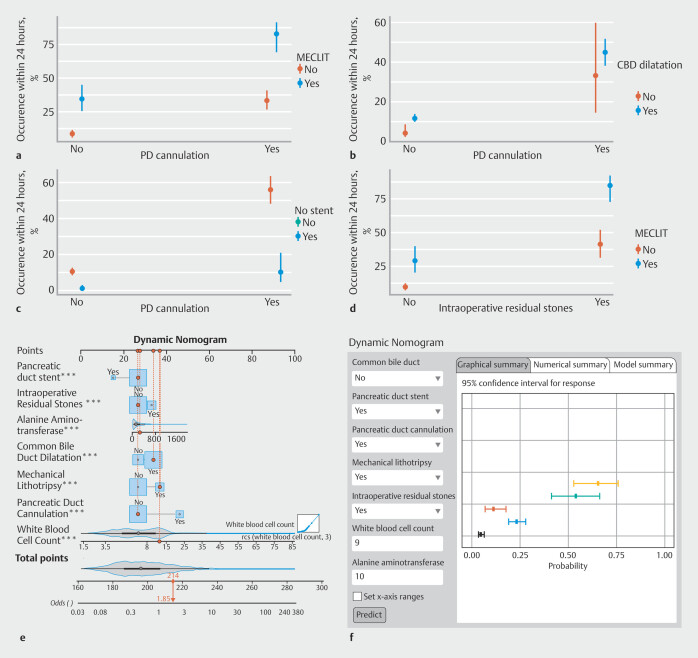
Interaction effect plots and web development of dynamic nomograms. **a** Interaction effect plot between mechanical lithotripsy and pancreatic duct cannulation. Mechanical lithotripsy significantly increased the 24-hour complication risk in patients who underwent guidewire insertion into the pancreatic duct. **b** Interaction effect plot between bile duct dilation and pancreatic duct cannulation. Pancreatic duct cannulation in patients who underwent bile duct dilation compounded the risk of complications. **c** Interaction effect plot between pancreatic duct stenting and pancreatic duct cannulation. Patients undergoing pancreatic duct cannulation, pancreatic duct stenting drastically reduced the complication rate, reflecting its protective effect. **d** Interaction effect plot between mechanical lithotripsy and intraoperative residual stones. Incomplete stone removal during mechanical lithotripsy was linked to increased complication rates. **e** Dynamic nomogram for predicting 24-hour post-ERCP complication risk. **f** Web-based interactive nomogram and a display of individual predictions with 95% confidence intervals. MECLIT, mechanical lithotripsy; CBD, common bile duct; PD, pancreatic duct; IRS, intraoperative residual stones.

ROC curves and performance metrics of the models in the external test set were subsequently generated ( Fig. 3 **a,b** ). AUC values for the RF, XGBoost, KNN, SVM, and NB models were 0.767, 0.689, 0.564, 0.726, and 0.706, respectively, indicating a performance drop with respect to the validation set. Among the five models, the RF model performed the best. ROC curve analysis revealed that AUC values of the univariate to multivariate LR and LASSO models in the external test set were 0.819 and 0.814. AUCs for the forward and backward stepwise regression were 0.812 and 0.806, respectively ( Fig. 3 **c** ). Other clinical evaluation indicators were also constructed ( Fig. 3 **d-f** ). These results indicate that the univariate to multivariate LR model outperformed the other models in the external test set. The SEN, SPE, ACC, positive predictive value (PPV), and negative predictive value (NPV) were 0.655, 0.835, 0.807, 0.423, and 0.929, respectively. The Hosmer–Lemeshow goodness-of-fit test for the model yielded *P* = 0.093, indicating that it had good calibration. In summary, the LR model was selected for further risk stratification. Interaction effect plots were used to analyze the interplay between key risk factors for post-ERCP complications. These findings highlight the complex interactions between the identified risk factors ( Fig. 4 **a-d** ). To further stratify risk with the LR model, the threshold corresponding to the maximum Youden index was chosen as the cut-off value. At this optimal cut-off (0.35), the SEN, SPE, ACC, PPV, and NPV were 0.710, 0.740, 0.729, 0.61, and 0.817, respectively. Patients whose predicted risk was < 35% were classified into the low-risk group, whereas those whose predicted risk was ≥ 35% were categorized into the high-risk group. A dynamic nomogram was developed using the seven key features identified in the model. For the continuous variables WBC and ALT, RCS curves were constructed to evaluate their nonlinear relationships with the outcome ( **Supplementary Fig. 6** ). WBC was identified as a strongly nonlinear variable; therefore, three knots were specified within its range for modelling, which was subsequently used to construct a web-based dynamic nomogram. On this basis, an online calculator was constructed in Shiny to predict likelihood of complications occurring within 24 hours after ERCP and to perform risk stratification ( https://borujin.shinyapps.io/dynnomapp/ ). The online calculator ( Fig. 4 **e,f** ) enables patient-specific risk prediction by integrating seven model predictors: five categorical variables (bile duct dilation, pancreatic stent, pancreatic cannulation, mechanical lithotripsy, residual stones) presented as binary options and two continuous variables requiring unit-specified inputs (WBC in ×10⁹/L, ALT in U/L). To further validate predictive performance of the univariate to multivariate LR model, an additional set of retrospectively collected data from 199 patients was included for secondary validation. In this additional dataset, the model achieved an AUC of 0.805, indicating strong discriminative ability. Moreover, the SEN, SPE, ACC, PPV, and NPV were 0.714, 0.799, 0.784, 0.431, and 0.929, respectively ( **Supplementary Fig. 7** ).

## Discussion

ESGE clearly states that difficult biliary cannulation, repeated pancreatic duct cannulation, biliary sphincter balloon dilation, incomplete stone removal, intraoperative bleeding, inadequate biliary drainage, and bile duct dilation are significant risk factors for post-ERCP complications 5 . Persistent bile duct dilation may elevate intraluminal pressure, inducing ischemic injury to the ductal wall and triggering localized inflammatory responses. Incomplete stone removal can perpetuate mechanical irritation of the biliary epithelium, potentially leading to recurrent stricture formation. Previous studies have established guidewire insertion into the pancreatic duct as a significant risk factor for PEP. Therefore, during difficult initial biliary cannulation, applying a guidewire-assisted technique demonstrates higher success rates while reducing PEP incidence and need for precut sphincterotomy compared with contrast-assisted methods 12 . Guidewire insertion into the pancreatic duct may induce mechanical injury, which can directly trigger pancreatic enzyme activation and pro-inflammatory cytokine cascades, ultimately leading to clinical pancreatitis 13 . In addition, pancreatic duct cannulation may increase intraductal hydrostatic pressure, leading to pancreatic juice reflux, which exacerbates postoperative inflammation and tissue edema. Leakage of pancreatic juice into surrounding tissues can further damage peripancreatic structures, thereby inducing pancreatitis 14 15 . Mechanical lithotripsy is useful for managing larger stones but can cause physical damage to the bile duct wall, potentially leading to localized inflammation and edema 16 , which increases risk of complications. Compared with balloon dilation for stone removal, mechanical lithotripsy is also associated with a higher incidence of infections and pancreatitis 17 . A sharp increase in leukocyte count before or after the ERCP procedure may indicate biliary infection or pancreatic duct injury. Similarly, abnormally elevated ALT preoperatively is often linked to severe obstruction of the CBD or concurrent inflammation of the pancreatic and biliary system.

Numerous studies have demonstrated that pancreatic duct stenting significantly reduces risk of PEP in high-risk patients, particularly in cases involving a difficult biliary cannulation or unintentional guidewire insertion into the pancreatic duct 18 19 .

Several clinical studies have investigated outpatient ERCP procedures to different extents. Elfant conducted a prospective analysis of safety, success, and postoperative complication rates of outpatient ERCP in patients with CBD stones 20 . The study included 97 patients who were properly observed and followed up postoperatively. Only one patient required hospitalization; the remaining patients were discharged safely, demonstrating that outpatient ERCP for stone removal does not compromise safety or success rates. Similarly, Marcal 21 conducted a prospective study involving 195 patients who underwent outpatient ERCP, 116 of whom required the procedure. Seven patients were hospitalized due to postoperative complications and eight were readmitted after discharge. There was no significant difference in outcomes between these patients, further supporting the safety of outpatient ERCP. Katsinelos 22 conducted a prospective study involving 600 patients, dividing them into groups younger than and older than 80 years. The study revealed no significant difference in postoperative complication rates between the two groups, suggesting that age alone should not be a contraindication to outpatient ERCP. These findings collectively support safety and efficacy of outpatient ERCP, even in older populations, provided that appropriate patient selection and postoperative monitoring are conducted. Although these clinical studies provide important evidence of the safety of outpatient ERCP, a systematic predictive model for accurately assessing and identifying patients suitable for outpatient procedures is lacking. This study introduces the first validated prediction model for selecting choledocholithiasis patients suitable for day-surgery ERCP.

Focusing on 24-hour outcomes essential for discharge decisions, the model was developed through comprehensive comparison framework. We provide a publicly accessible online calculator for real-time risk assessment, translating research into practice by offering a pragmatic approach to standardize and safely expand day-surgery ERCP for bile duct stone management.

All models showed performance decline during external validation. This primarily results from inherent heterogeneity introduced by the external validation sets, which originated from different medical centers and time periods compared with the training cohort, exhibiting variations in patient demographics, endoscopic techniques, perioperative management protocols, and data recording practices. The more pronounced performance decline observed with complex machine learning models indicates their heightened susceptibility to overfitting specific patterns and noise in the development data. In contrast, the relative resilience of the LR model to this performance decay underscores its robustness. Despite this expected decrease, LR maintained AUCs above 0.8 in both external validation sets, demonstrating preserved discriminatory power that we consider clinically applicable for risk stratification in day-surgery ERCP.

A nomogram was created in this study to visualize independent contributions of various the risk factors to development of post-ERCP complications. Regarding the cutoff value, patients with a predicted risk score > 0.35 were deemed high risk and recommended for inpatient observation or hospitalization, whereas those with a score < 0.35 could be safely discharged.

The methodology rigorously characterizes the nonlinear relationships among continuous variables. This approach facilitates development of a highly precise and reliable dynamic nomogram, accompanied by a web-based predictive tool. This study has several limitations. First, the model includes a limited number of variables, which may affect the reliability of the results. Second, our model's applicability is specifically limited to choledocholithiasis patients with intact gallbladders and should not be extended to post-cholecystectomy cases or other ERCP indications—including malignant biliary obstruction, benign biliary strictures, and sphincter of Oddi dysfunction—because these patient groups and conditions demonstrate fundamentally distinct risk profiles and underlying pathophysiological mechanisms. Third, the high NBD utilization in our cohort may limit the model's generalizability. Although considered initially, this variable was not retained in the final model. Future validation in stent-preferring populations is essential to ensure broad clinical applicability. Fourth, the model's cut-off value of 0.35 resulted in a high false-positive rate and, thus, could lead to unnecessary hospitalizations or interventions. Future studies should address these limitations by increasing the sample size and incorporating additional predictive variables to increase accuracy and reliability of the model. Fifth, this study's scope is limited to classical machine learning algorithms, excluding newer architectures with potentially distinct performance. Future studies should expand the model spectrum to validate and enhance our approach. The core innovation and strength of this study lie in developing the first clinical decision tool specifically designed for day-surgery ERCP pathways, moving beyond the generalized scope of traditional complication prediction models. Unlike existing ERCP-related prediction models that primarily focus on algorithm optimization, our work directly addresses the crucial clinical question of “which patients are suitable for 24-hour discharge”, thereby advancing from mere prediction to clinical decision support.

## Conclusions

The LR model which identified mechanical lithotripsy, pancreatic duct cannulation, bile duct dilation, residual stones during the procedure, white blood cell count, alanine aminotransferase (ALT) level and pancreatic duct stent placement can effectively predict risk of post-ERCP complications in patients with CBD stones, providing accurate guidance for risk stratification. This study contributes to development and management of outpatient ERCP procedures, ensuring safer and more efficient clinical decision-making.

## Supplementary Material

Supplementary Material Zusatzmaterial
