# Supplementary material for: Identifying high-risk patients having ERCP as a day surgery with an online prediction platform: Multicohort validation of a machine learning model
Source: Endosc Int Open. 2025 Dec 16;13:a27331387. doi: 10.1055/a-2733-1387 (PMC12817187; doi:10.1055/a-2733-1387)

Thieme

Supplementary Table 1 Baseline characteristics after adaptive synthetic sampling.

**Variables**	**Overall**<br>**N = 2201**	**No complications within 24 hours**<br>**N = 1400**	**Complications within 24 hours**<br>**N = 801**	***P* value**
Gender (%)				
Female	990 (45.0)	629 (44.9)	361 (45.1)	0.949
male	1211 (55.0)	771 (55.1)	440 (54.9)	
Common bile duct dilatation (%)				
No	609 (27.7)	457 (32.6)	152 (19.0)	< 0.001
Yes	1592 (72.3)	943 (67.4)	649 (81.0)	
Common bile duct obstruction (%)				
No	1445 (65.7)	952 (68.0)	493 (61.5)	0.002
Yes	756 (34.3)	448 (32.0)	308 (38.5)	
Multiple stones (%)				
No	861 (39.1)	568 (40.6)	293 (36.6)	0.065
Yes	1340 (60.9)	832 (59.4)	508 (63.4)	
Bile duct stent (%)				
No	1328 (60.3)	861 (61.5)	467 (58.3)	0.14
Yes	873 (39.7)	539 (38.5)	334 (41.7)	
Coronary heart disease (%)				
No	2106 (95.7)	1346 (96.1)	760 (94.9)	0.161
Yes	95 (4.3)	54 (3.9)	41 (5.1)	
Chronic lung disease (%)				

No	2151 (97.7)	1375 (98.2)	776 (96.9)	0.043
Yes	50 (2.3)	25 (1.8)	25 (3.1)	
Intraoperative bleeding (%)				
No	2008 (91.2)	1283 (91.6)	725 (90.5)	0.367
Yes	193 (8.8)	117 (8.4)	76 (9.5)	
Pancreatic duct stent (%)				
No	2080 (94.5)	1334 (95.3)	746 (93.1)	0.033
Yes	121 (5.5)	66 (4.7)	55 (6.9)	
Difficult cannulation (%)				
No	1992 (90.5)	1285 (91.8)	707 (88.3)	0.007
Yes	209 (9.5)	115 (8.2)	94 (11.7)	
Pancreatic duct cannulation (%)				
No	1857 (84.4)	1262 (90.1)	595 (74.3)	< 0.001
Yes	344 (15.6)	138 (9.9)	206 (25.7)	
Epinephrine spray (%)				
No	1982 (90.0)	1274 (91.0)	708 (88.4)	0.049
Yes	219 (10.0)	126 (9.0)	93 (11.6)	
Electrocautery hemostasis (%)				
No	2193 (99.6)	1399 (99.9)	794 (99.1)	0.003
Yes	8 (0.4)	1 (0.1)	7 (0.9)	

Young female (< 45 years) (%)				
No	1958 (89.0)	1244 (88.9)	714 (89.1)	0.839
Yes	243 (11.0)	156 (11.1)	87 (10.9)	
Endoscopic Sphincterotomy (%)				
No	250 (11.4)	166 (11.9)	84 (10.5)	0.33
Yes	1951 (88.6)	1234 (88.1)	717 (89.5)	
Endoscopic papillary balloon dilation (%)				
No	1561 (70.9)	1026 (73.3)	535 (66.8)	0.001
Yes	640 (29.1)	374 (26.7)	266 (33.2)	
Periampullary duodenal diverticulum (%)				
No	1710 (77.7)	1101 (78.6)	609 (76.0)	0.157
Yes	491 (22.3)	299 (21.4)	192 (24.0)	
Mechanical lithotripsy (%)				
No	1772 (80.5)	1211 (86.5)	561 (70.0)	< 0.001
Yes	429 (19.5)	189 (13.5)	240 (30.0)	
Nasobiliary drainage (%)				
No	797 (36.2)	499 (35.6)	298 (37.2)	0.464
Yes	1404 (63.8)	901 (64.4)	503 (62.8)	
Intraoperative residual stones (%)				
No	1702 (77.3)	1155 (82.5)	547 (68.3)	< 0.001

Yes	499 (22.7)	245 (17.5)	254 (31.7)	
Hypertension (%)				
No	1743 (79.2)	1103 (78.8)	640 (79.9)	0.535
Yes	458 (20.8)	297 (21.2)	161 (20.1)	
Diabetes mellitus (%)				
No	2017 (91.6)	1295 (92.5)	722 (90.1)	0.054
Yes	184 (8.4)	105 (7.5)	79 (9.9)	
Age (median [IQR])	62.00 [49.20, 72.00]	62.00 [49.00, 73.00]	62.00 [50.53, 70.45]	0.541
MRCPDI (median [IQR])	10.60 [9.00, 13.00]	10.00 [8.28, 13.00]	11.00 [9.00, 13.00]	0.008
DBIL (median [IQR])	17.20 [6.10, 58.88]	15.35 [5.70, 53.15]	22.86 [6.62, 62.60]	0.002
Height (median [IQR])	1.65 [1.60, 1.72]	1.65 [1.60, 1.72]	1.65 [1.60, 1.72]	0.729
TG (median [IQR])	1.33 [0.95, 2.06]	1.35 [0.95, 2.04]	1.32 [0.95, 2.09]	0.47
Operative time (median [IQR])	40.00 [30.00, 50.00]	40.00 [28.00, 50.00]	40.00 [30.00, 50.00]	0.613
BILMAX (median [IQR])	0.80 [0.50, 1.00]	0.80 [0.50, 1.00]	0.80 [0.50, 1.00]	0.39
HGB (median [IQR])	139.54 [126.00, 152.96]	140.00 [125.00, 153.00]	139.53 [127.24, 151.00]	0.759
PLT (median [IQR])	177.00 [126.92, 234.00]	184.00 [131.75, 235.00]	167.89 [119.29, 229.64]	0.005
ALB (median [IQR])	40.30 [36.40, 44.00]	40.20 [36.00, 44.20]	40.59 [36.90, 43.79]	0.406
BMI (median [IQR])	22.86 [20.76, 25.16]	22.77 [20.70, 25.26]	22.97 [20.85, 25.02]	0.472

WBC (median [IQR])	6.79 [5.04, 9.94]	5.94 [4.67, 8.20]	9.47 [5.91, 12.22]	< 0.001
Blood amylase (median [IQR])	58.25 [43.00, 85.00]	58.70 [44.00, 86.00]	58.10 [43.00, 81.00]	0.852
ALP (median [IQR])	197.44 [123.02, 350.20]	196.00 [123.00, 347.00]	203.82 [124.66, 376.40]	0.386
YGT (median [IQR])	296.31 [121.40, 597.50]	294.35 [110.22, 556.22]	315.82 [135.32, 650.40]	0.002
ALT (median [IQR])	115.00 [42.00, 251.00]	96.70 [38.00, 218.25]	151.88 [56.25, 312.30]	<0.001

ALB, albumin; ALP, alkaline phosphatase; ALT, alanine aminotransferase; AST, aspartate aminotransferase; BILMAX, biliary stone maximum diameter; BMI, body mass index; DBIL, direct serum bilirubin; MRCPDI, inner duct diameter on magnetic resonance cholangiopancreatography; PLT, platelet; TG, triglyceride; WBC, white blood cell; YGT, gamma-glutamyl transferase.

Supplementary Table 2 Parameter tuning tables for five machine learning models with ten-fold cross-validation.

Model	Optimal parameter
RF	n_trees = 500, min_node_size = 1<br>Mtry = 1, splitrule = gini
XGBoost	eta = 0.1, max_depth = 9, gamma = 0, subsample = 0.8<br>colsample_bytree = 0.6, min_child_weight = 1, nrounds = 100
KNN	k = 3
SVM	sigma = 0.1, c = 10
NB	usekernel = TRUE, FL = 0, adjust = 0.5

KNN, K-nearest neighbors; NB, naïve Bayes; RF, random forest; SVM, support vector machine; XGBoost, eXtreme gradient boosting.

Supplementary Figure 1. Study design and patient flow chart in our study.

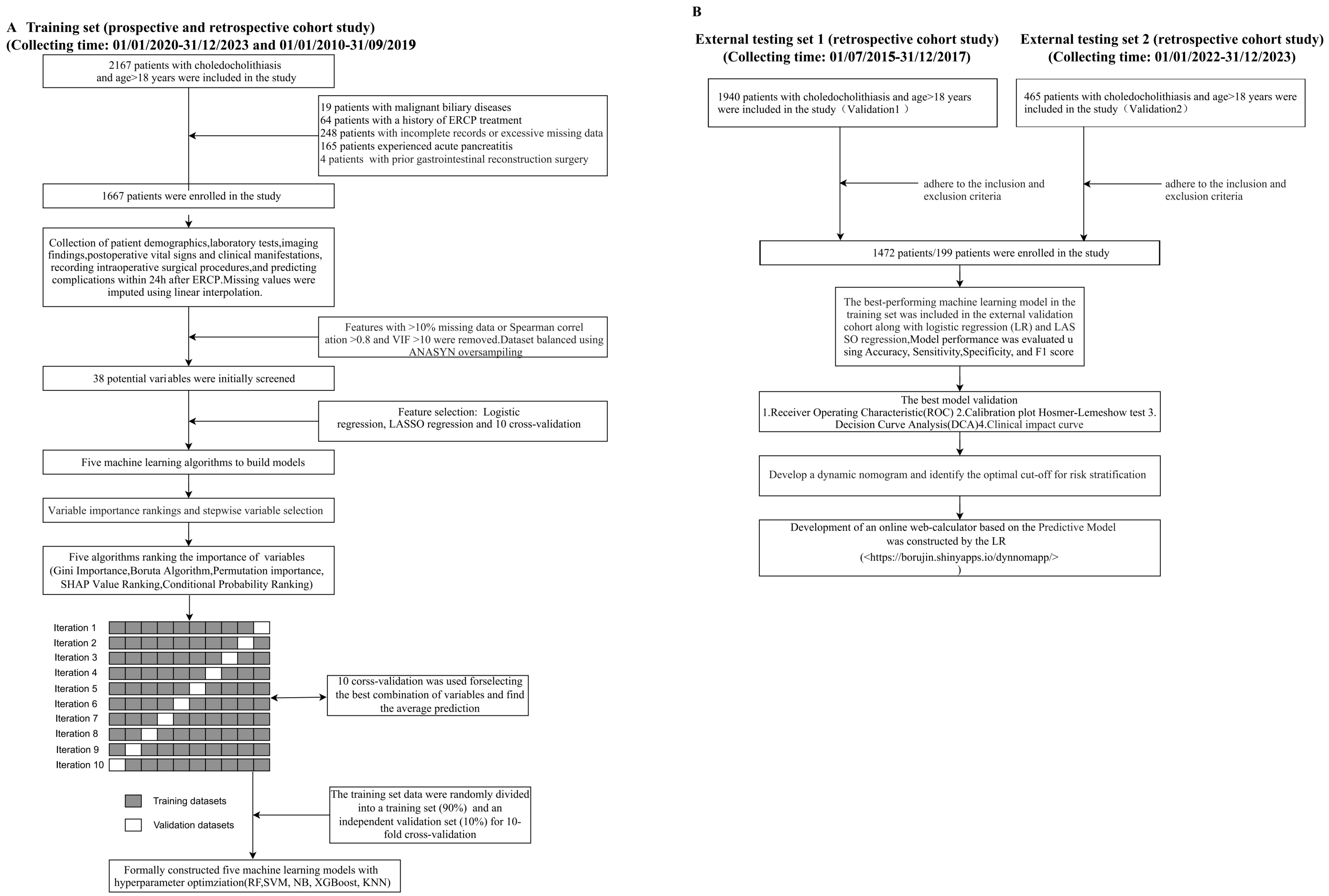

Supplementary Figure 2. Spearman correlation coefficient plot and Variance Inflation Factor (VIF) plot.

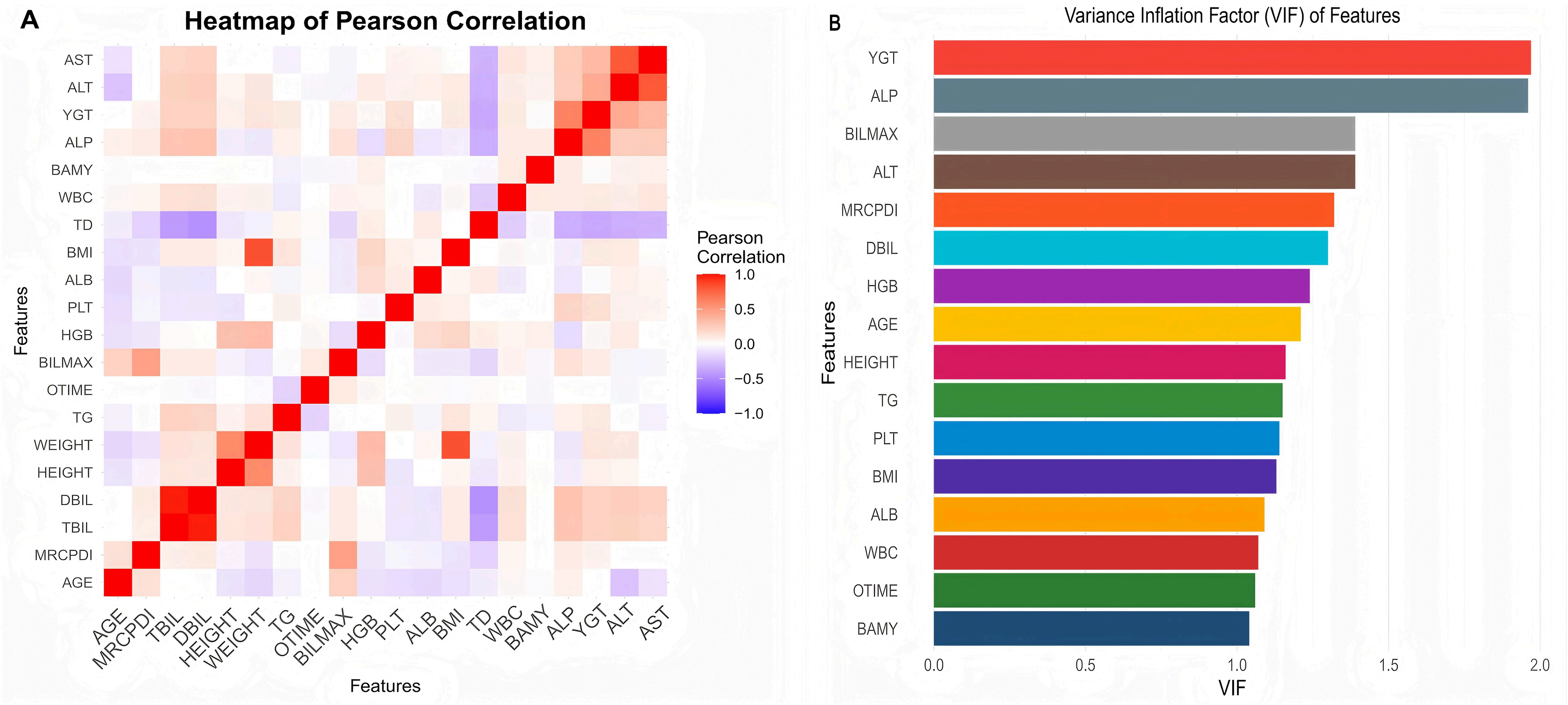

Supplementary Figure 3. Oversampling-induced changes in feature variable frequency and data distribution.

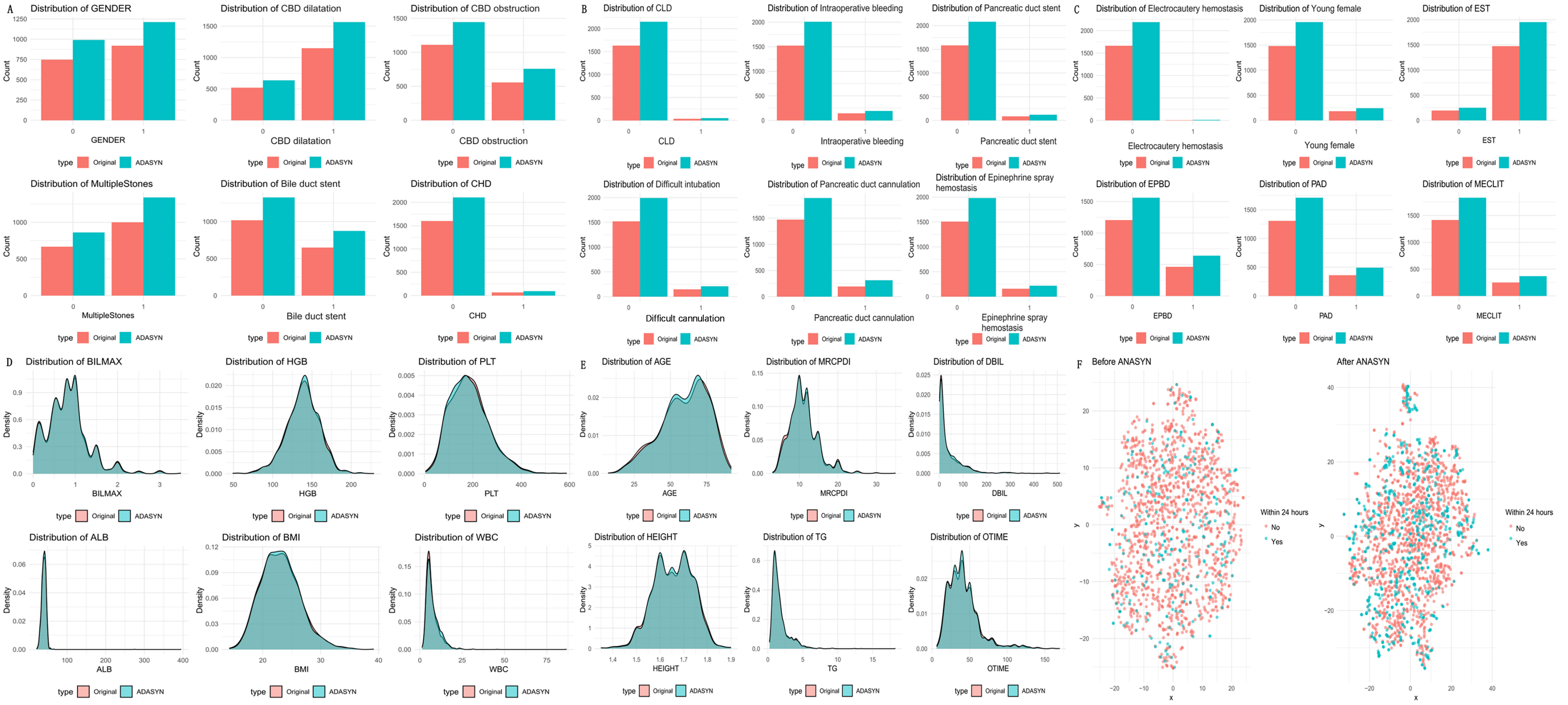

Supplementary Figure 4. Variable importance ranking plots and optimal feature trend line plot.

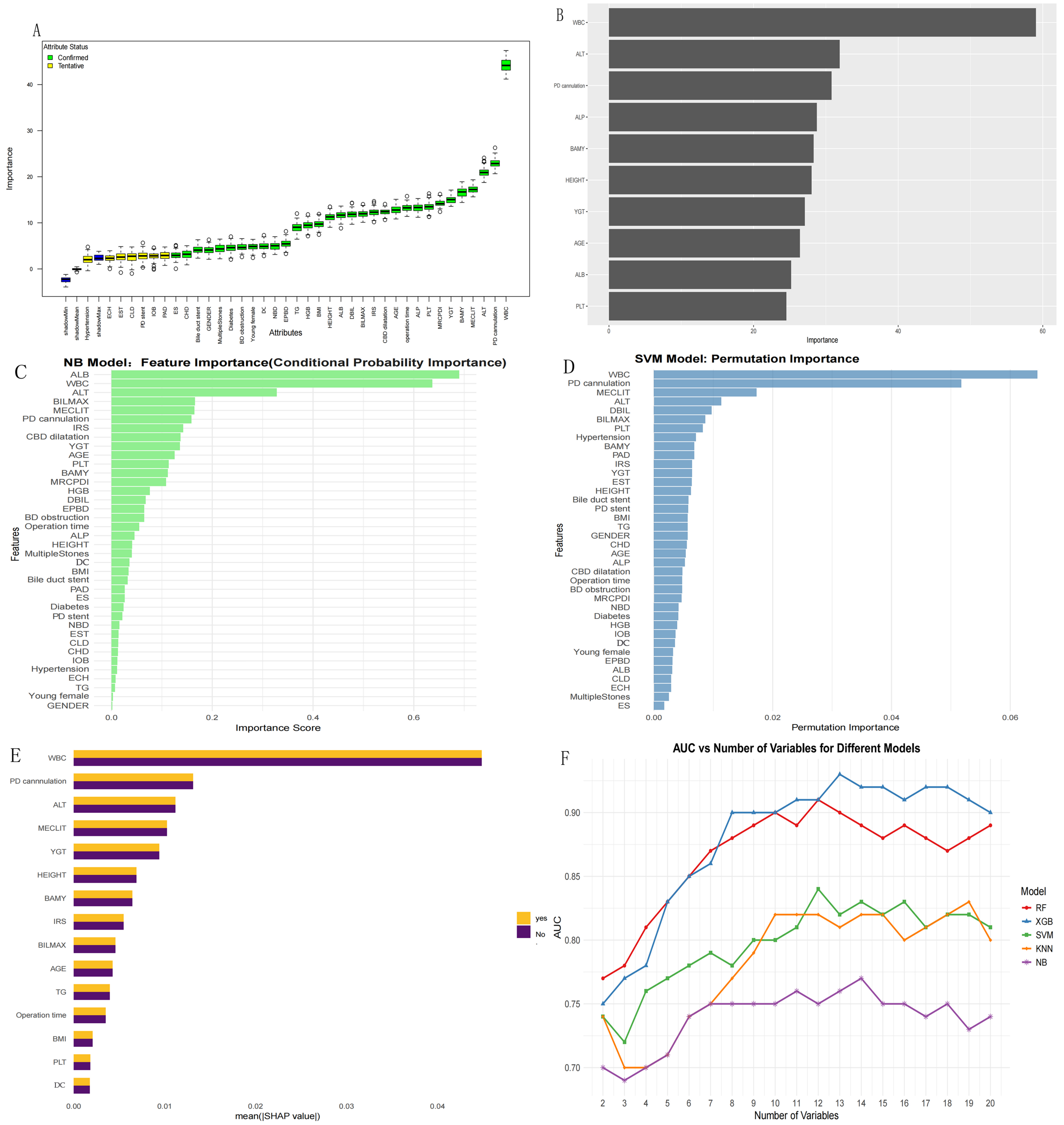

Supplemenary Figure 5. Parameter tuning plots for five machine learning models with ten-fold cross-validation.

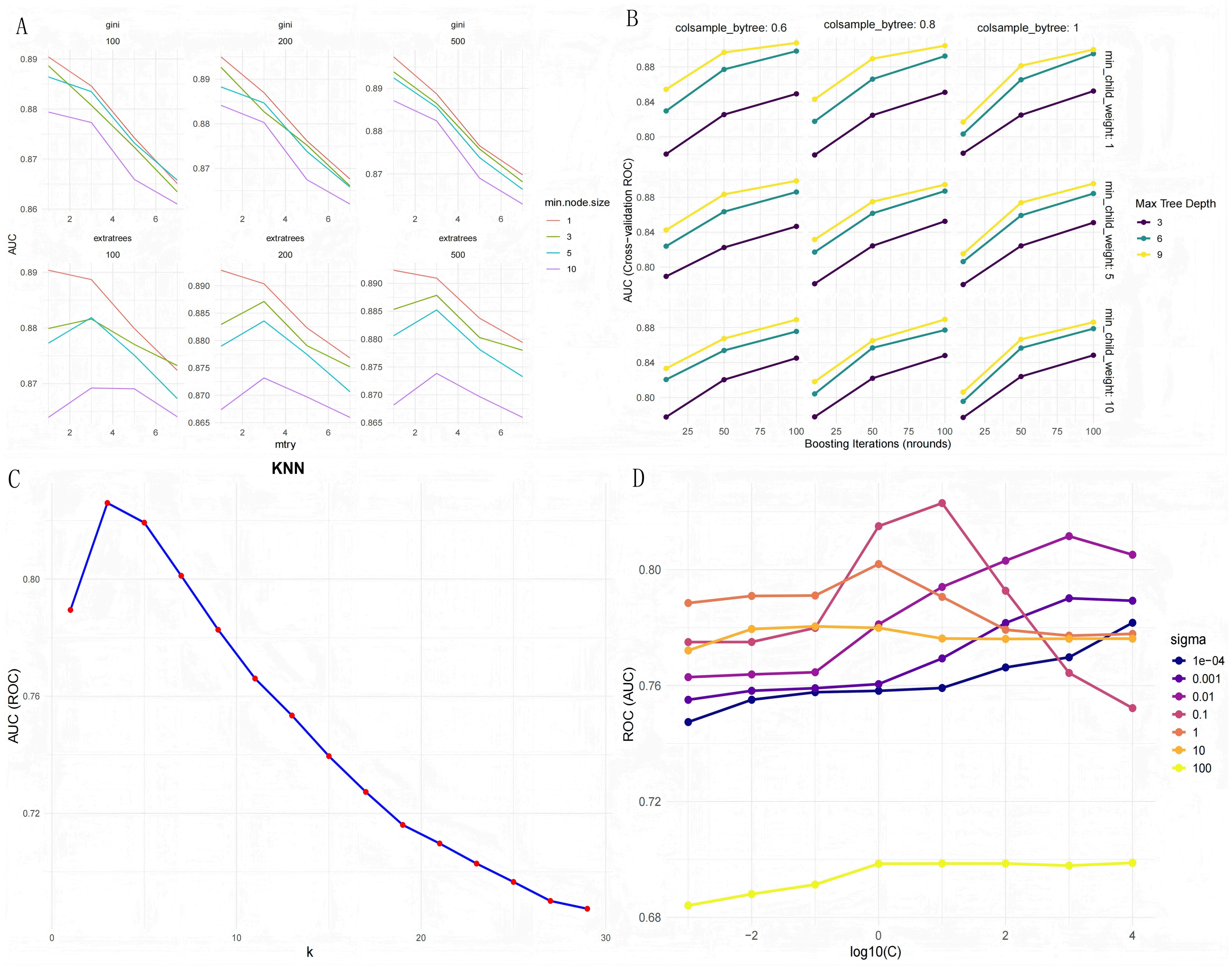

Supplementary Figure 6. Restricted Cubic Spline Curve for Modeling Continuous Variables.

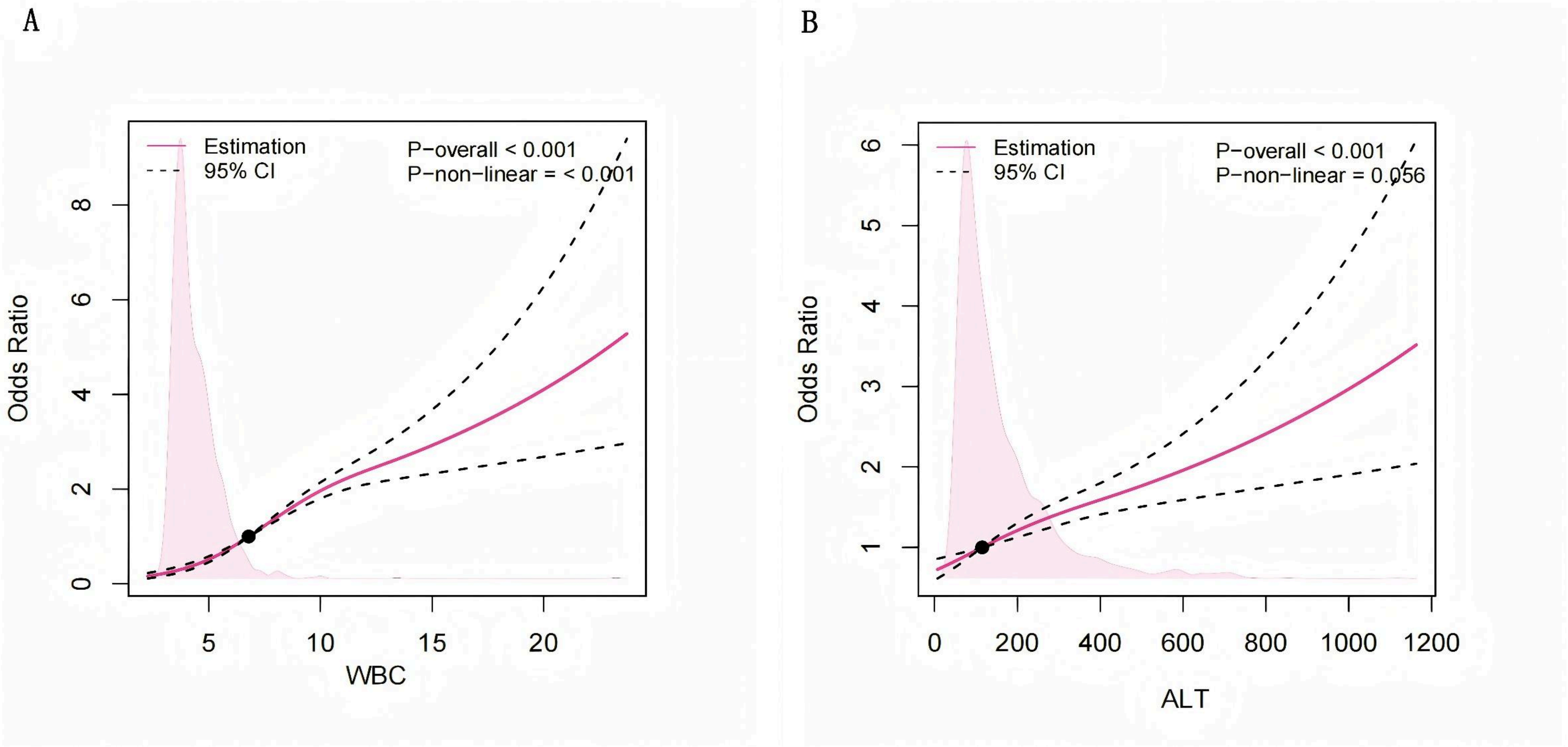

Supplementary Figure 7. Evaluation metrics for Univariate to Multivariate logistic regression Model on External Testing Set Two.

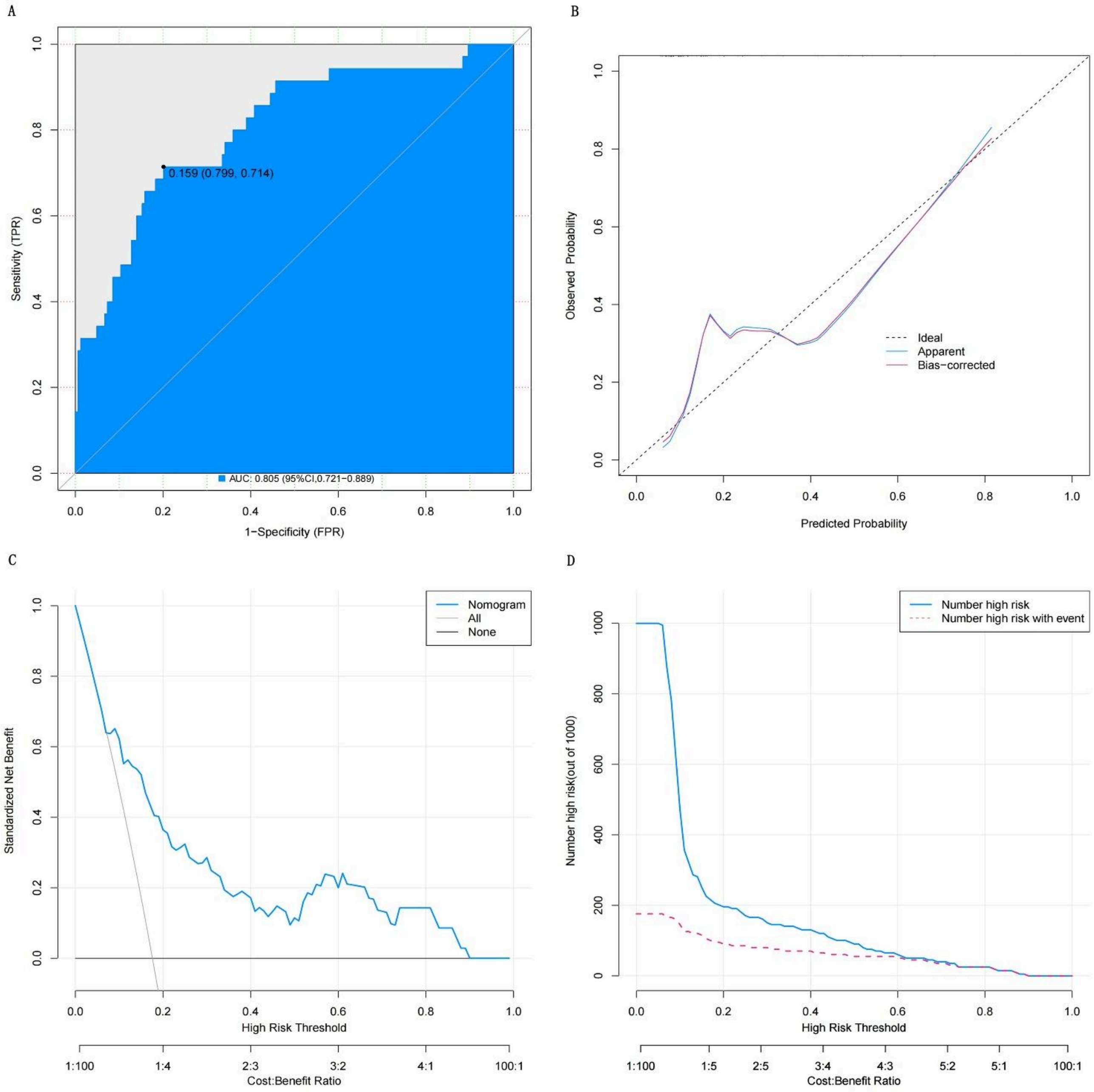

Supplement: Supplementary file 1 — Supplementary Material [file 10-1055-a-2733-1387_27356127.pdf]
